# An All-in-One Vehicle Type and License Plate Recognition System Using YOLOv4

**DOI:** 10.3390/s22030921

**Published:** 2022-01-25

**Authors:** Se-Ho Park, Saet-Byeol Yu, Jeong-Ah Kim, Hyoseok Yoon

**Affiliations:** 1 Contents Convergence Research Center, Korea Electronics Technology Institute, Mapo-gu, Seoul 03924, Korea; sehopark@keti.re.kr (S.-H.P.); sbyu@keti.re.kr (S.-B.Y.); kpkk1518@keti.re.kr (J.-A.K.); 2 Division of Computer Engineering, Hanshin University, Osan-si 18101, Korea

**Keywords:** license plate detection, license plate recognition, make and model recognition, vehicle type detection, YOLOv4

## Abstract

In smart surveillance and urban mobility applications, camera-equipped embedded platforms with deep learning technology have demonstrated applicability and effectiveness in identifying various targets. These use cases can be found in a variety of contexts and locations. It is critical to collect relevant data from the location where the application will be deployed. In this paper, we propose an integrated vehicle type and license plate recognition system using YOLOv4, which consists of vehicle type detection, license plate detection, and license plate character detection to better support the context of Korean vehicles in multilane highway and urban environments. Using our dataset of one to four multilane images, our system detected six vehicle classes and license plates with mAP of 98.0%, 94.0%, 97.1%, and 84.6%, respectively. On our dataset and a publicly available open dataset, our system demonstrated mAP of 99.3% and 99.4% for the detected license plates, respectively. From 4K high-resolution images, our system was able to detect minuscule license plates as small as 100 pixels wide. We believe that our system can be used in densely populated regions to address the high demands for enhanced visual sensitivity in smart cities and Internet-of-Things.

## 1. Introduction

**Figure 1:**
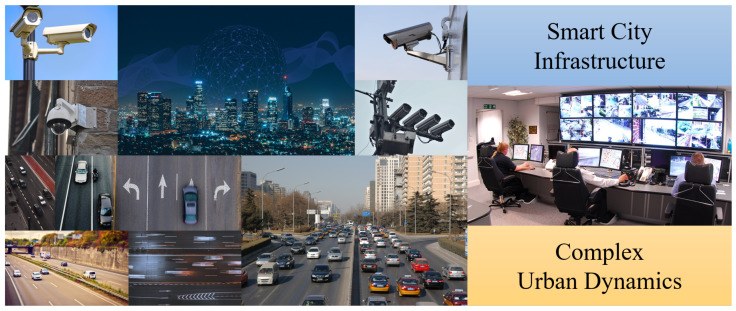
Pervasive visual sensors capture complex urban dynamics in smart cities.

Computer vision applications automate repetitive tasks that require the human ability and attention to continuously monitor and make timely decisions. A profusion of such applications has been developed to detect, identify, and track various objects of interest. Recent advancements in smart city technologies [1] have enabled a plethora of visual sensors to be installed in the intelligent environment and smart infrastructure, such as closed-circuit television (CCTV), visual sensor networks [2], smart surveillance [3], intelligent traffic systems [1,4], security cameras, and black boxes in vehicles. A series of state-of-the-art deep learning techniques for challenging computer vision problems [5] can detect and identify a vast number of diverse objects across categories on a grand scale. Individuals and their vehicles are significant subjects of interest in large cities and metropolitan regions, which smart cameras try to recognize. A large number of license plate recognition (LPR) [6,7,8] and make and model recognition (MMR) [9,10,11] systems have been developed to relieve human operators of the tedious task of explicitly detecting, identifying, and recognizing a wide range of cars, as illustrated in Figure 1.

In this regard, we are particularly motivated to recognize modern Korean vehicle types (VT) and Korean license plates (LP) in areas with high vehicle density in South Korea. The number of cars registered in South Korea exceeded 24 million in 2020, according to the Korean Statistical Information Service, which is roughly equivalent to one car per 2.19 people or 456.6 cars per 1000 people. Furthermore, Seoul (i.e., the capital and largest metropolis of South Korea) is one of the most surveilled cities in the world, boasting 77,564 cameras for 234 square miles or 331.94 cameras per square mile (source: https://www.comparitech.com/vpn-privacy/the-worlds-most-surveilled-cities/, accessed on 7 December 2021). In an ever-increasingly complex urban environment, we propose an all-in-one system named KVT-LPR which stands for Korean vehicle type and license plate recognition system, capable of identifying both VTs and LPs in the same processing pipeline.

Our contributions in this paper are as follows.

- We propose a two-phase architecture based on YOLOv4 [12] for detecting vehicle types and recognizing Korean LPs in one pipeline.
- We collect and build a custom dataset for various Korean vehicle types and LPs captured from multilanes to train and validate two custom detectors in the KVT-LPR.
- We show that the KVT-LPR effectively detects small license plates from 4K high-resolution input images, which is an enhancement over previous detectors.
- We demonstrate the feasibility and applicability of the KVT-LPR’s practically deployed detection performance in different settings across two datasets (i.e., a custom dataset and a publicly open dataset) and two target platforms (i.e., from a high-end to an embedded solution).

## 2. Related Work

There have been a series of attempts to build faster and more accurate LPR systems. In recent years, deep learning-based approaches, such as single shot detector (SSD) [13] and You Only Look Once (YOLO)-based models [12,14,15,16], have been used to detect and recognize LPs. YOLO was first designed to provide fast detection speed, but it had low accuracy [14]. Despite the fact that YOLOv2 enhanced the speed and accuracy of object identification over its predecessor [15], the SSD still outperformed for smaller objects. YOLOv3’s accuracy has improved since then, but its detection speed has slowed down [16]. YOLOv4 has improved performance in both speed and accuracy compared to YOLOv3 [12]. Hendry and Chen tweaked the original YOLO to create an automatic license plate recognition (ALPR) system that had a detection accuracy of 98.22% and a recognition accuracy of 78.22% [17]. Laroca et al. developed an ALPR system based on YOLO that outperformed previous systems with a recognition rate of 96.9% when tested on public datasets [18]. Castro-Zunti et al. presented an SSD-based LPR system that accurately recognized 96.23% of the Caltech Cars dataset and 99.79% of the UCSD-Stills dataset [19].

There are several related LPR systems targeting Korean LPs and sharing similar approaches. Han et al. used the cascade structure with AdaBoost learning to offer a real-time LPR identification method for high-resolution videos [20]. Park et al. developed a multinational LPR system that recognizes multiple Korean LP styles (i.e., single-line, double-line, various layout formats) using the K-nearest neighbors method [21]. By adding spatial pyramid pooling to YOLOv3, Kim et al. developed a multiscale vehicle detection that outperformed other detectors [22]. For recognizing multinational LPs, including Korean LPs, Henry et al. presented an ALPR system based on YOLOv3 [23]. LP detection, unified character recognition, and multinational LP layout detection were all included in their system’s architecture. Initially, they have collected and made public their own Korean automobile plate dataset, known as KarPlate. However, due to legal issues, the dataset is no longer available. Sung et al. showed Korean LP identification performance on the NVIDIA Jetson TX2 board with their custom KETI-ALPR dataset that is not open to the public using YOLOv3, YOLOv4, and SSD [24]. To recognize Korean car types, Kim et al. evaluated faster-RCNN, YOLOv4, and SSD object identification approaches [25]. Their findings revealed that YOLOv4 outperformed SSD and faster-RCNN in terms of F1 score, precision, recall, and mAP. To deal with the problem of data sparsity in the training stage, Han et al. synthesized LPs using an ensemble of generative adversarial networks (GAN) [26]. Wang et al. developed a Korean LPR approach using deep learning and KarPlate dataset (when the dataset was still available) to recognize LPs under various conditions (i.e., fog and haze) [27]. Lim and Park proposed an AI machine learning system that can use CCTV images to check illegally parked cars with the LPR function [28].

In contrast to prior research, this study investigates the application of YOLOv4 for LPR and vehicle type recognition in the Korean environment with multilanes and high-resolution cameras. Table 1 compares previous studies in terms of their approaches, datasets, and system support features. Our system aims to better support the Korean context by using multilanes images collected from high-resolution cameras. The size of LPs will be small in high-resolution images. We employ YOLOv4 to recognize small LPs and vehicle types and to show that its performance is embedded-platform-ready.

## 3. Proposed Methodology

The goal of a typical LPR system is to output numbers and characters on LPs as text. Similarly, a typical MMR system identifies the vehicle’s make and model from several candidates. Our goal was to create an LPR system that could identify Korean LPs and recognize a variety of Korean vehicle types as defined by the Korean vehicle classification criteria. We present an all-in-one Korean vehicle type and LP recognition system, named KVT-LPR, that employs YOLOv4 as the underlying object detector model.

**Figure 2:**
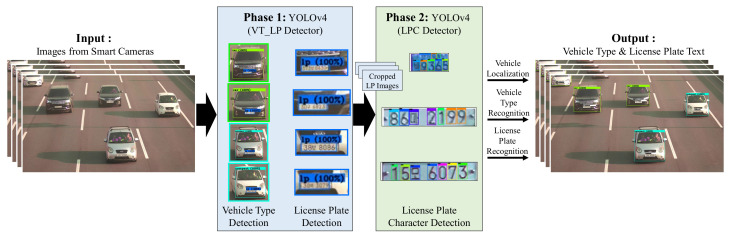
The overview of the KVT-LPR system.

Figure 2 shows the overview of our KVT-LPR using YOLOv4. The KVT-LPR aims to identify vehicle types and recognize license plates from high-resolution (i.e., 4K resolution) and multilane images (i.e., one to four lanes). The details of the KVT-LPR system, including YOLOv4-based object detector and data collection processes, are elaborated in the following subsections. Moreover, detailed procedures of the two custom detectors (VT_LP detector and LPC detector) are visually illustrated in Section 4.

### 3.1. YOLOv4-Based Vehicle Type and License Plate Recognition

The KVT-LPR system processes a high-resolution input image (i.e., 3840 × 2160) decoded from a high-resolution video. We collected real Korean vehicle types and LPs to build our custom dataset. Then, we used the custom dataset to train using YOLOv4 to build two custom detectors. The first detector is a VT_LP detector, which detects seven classes (i.e., six different Korean vehicle types and LPs) in the input image. The second detector is an LPC detector, which detects 68 different numbers and characters on Korean LPs. The character size of LPs is small in relation to the entire image on a high-resolution image, making character identification more challenging. To overcome this problem, we included an LP cropping procedure to the KVT-LPR, which gives the LPC detector segmented LP regions. In phase 1, vehicle types and occurrences of LPs are detected by the VT_LP detector. If LPs are found, the cropped LP image for each LP is passed into the LPC detector for phase 2. To summarize, the VT_LP detector is called first to detect vehicle types and LPs, followed by the LPC detector for each LP found. If the input image contains a large number of LPs, the KVT-LPR’s overall turnaround time multiplies.

### 3.2. Dataset Collection and Preprocessing

#### 3.2.1. Vehicle Types and LPs

**Figure 3:**
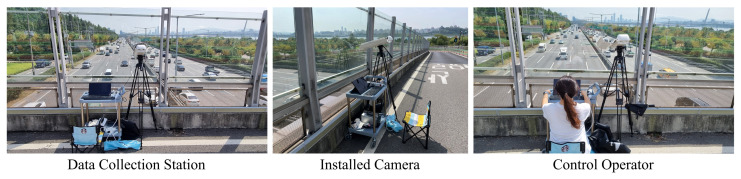
Data collection settings. Vehicles and LPs are captured using a camera fixed on a highway overpass with a residing operator controlling the data collection station.

We installed a camera on a highway overpass to manually record real traffic videos in order to collect various vehicle types and LP images that represent the context and environment of South Korea, as shown in Figure 3.

**Figure 4:**
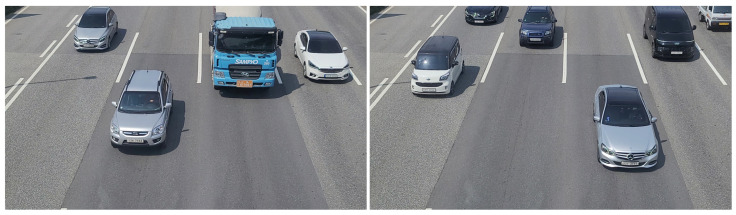
Examples of captured raw images from the installed camera.

The camera overlooking the highway (i.e., two-lane, three-lane, and four-lane) captured traffic videos at 3840 × 2160. We also recorded videos with a smartphone camera at 3840 × 2160. Images including one or more vehicles were extracted from the recorded video and used as training data for the custom detectors. Figure 4 shows examples of captured raw images that qualify for training uses.

**Figure 5:**
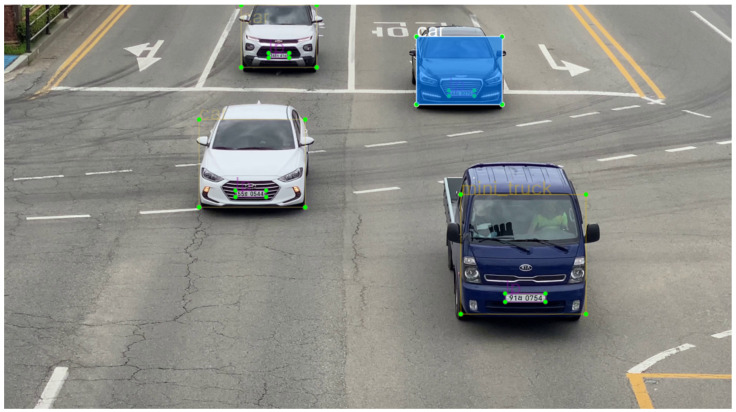
Labeling of vehicle types and license plates to be used for phase 1.

The collected dataset is manually labeled by using an open-source tool LabelImg (Tzutalin (TzuTa, Canada), LabelImg, Git code (2015). https://github.com/tzutalin/labelImg, accessed on 7 December 2021) to annotate bounding boxes on the target objects. For example, we annotated a bounding box on LPs and the front of a vehicle covering the front window and the bumper, as shown in Figure 5.

**Figure 6:**
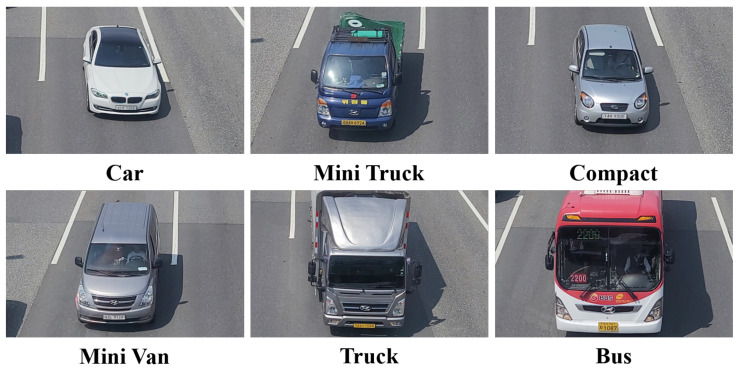
Classification of six vehicle types.

To label different vehicle types, we referenced a vehicle classification according to the vehicle size and passenger capacity used by the Korea Expressway Corporation (https://www.ex.co.kr/portal/usefee/selectUseFeeNList.do, accessed on 7 December 2021). We classified vehicles into six categories based on the vehicle size and passenger capacity. The smallest vehicles or compact cars were labeled as ‘compact’. Vehicles capable of holding nine or fewer passengers were labeled as ‘car’. Vehicles with a capacity of 25 or fewer passengers were labeled as ‘mini van’. Big vans with 25 or more passengers were labeled as ‘bus (big van)’. Smaller two-axle freight vehicles were labeled as ‘mini truck’, and three-or-more-axle freight vehicles were labeled as ‘truck’. The six vehicle types we labeled in our dataset are shown in Figure 6. Table 2 shows the collected dataset of six vehicle types and LPs.

#### 3.2.2. LP Numbers and Characters

**Figure 7:**
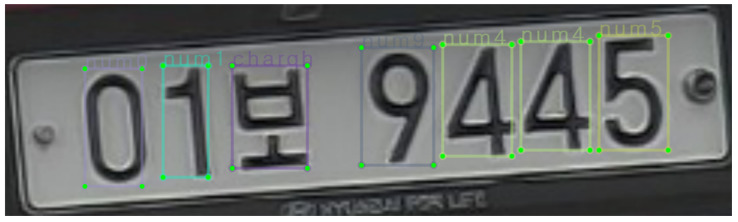
Labeling of LP numbers and characters to be used for phase 2.

The recorded videos were also used to manually label Korean LP numbers and characters. We also took additional pictures of LPs with a smartphone camera. LP areas were segmented and used as training data from these sources. In the case of LPs, a bounding box was drawn over the four vertices of an LP. Furthermore, bounding boxes were annotated on each number or character on LPs, as shown in Figure 7.

**Figure 8:**
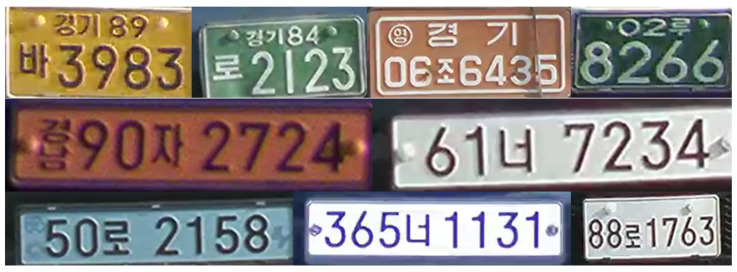
Different Korean LP styles, including single- and double-line LPs containing Korean characters and numbers listed in Table 3 and Table 4.

Over 60,000 occurrences of Korean LP numbers and characters were collected and grouped into 68 classes (i.e., numbers 0 to 9: class 0 to 9, 41 Korean characters: class 10 to 50, and 17 local area prefixes: class 51 to 67). Figure 8 shows different Korean LP styles, including single-line and double-line LPs. Area prefixes and predesignated Korean characters can be found on older LPs and special-purpose vehicles. Table 3 and Table 4 show the collected dataset for Korean LP numbers and characters. Note that we were not able to collect all LP characters, and numerous local area prefixes were left out (highlighted in gray in Table 4).

## 4. Experiments

To evaluate the feasibility and effectiveness of the KVT-LPR system, we evaluated the KVT-LPR system’s capability of detecting small LPs, detection speed, the performance of vehicle type detection, and the performance of LPR.

### 4.1. Implementation

To implement our proposed KVT-LPR system, we used YOLOv4 [12] as the underlying object detector. We used an open-source darknet framework to train YOLOv4 to detect our custom set of classes (i.e., vehicle types, LP, and LP characters). We had previously experimented with several image input sizes before settling on a 256 × 256 image input size for YOLOv4 [24]. We discovered a considerable performance decrease on the lower-end embedded platform, despite the fact that a bigger input size, such as 608, increased accuracy.

**Figure 9:**
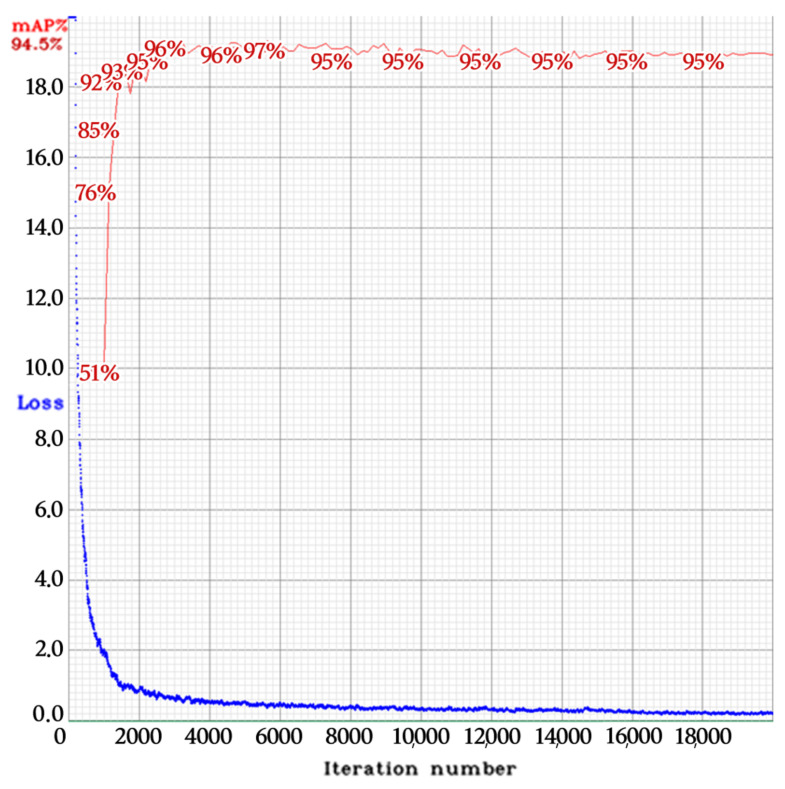
Training loss (blue) and mAP @ 0.5 (red) of the VT_LP detector in phase 1.

**Figure 10:**
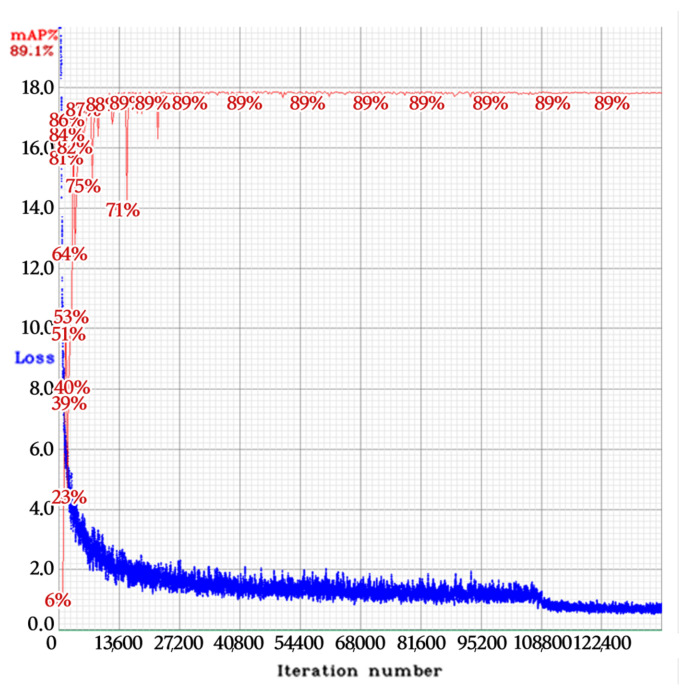
Training loss (blue) and mAP @ 0.5 (red) of the LPC detector in phase 2.

Figure 9 and Figure 10 show the training loss and the mean average precision at 50% intersection-over-union threshold (mAP @ 0.5). For the VT_LP detector, the collected dataset was used as 70% train, 17.5% validation, and 12.5% test sets for each class. For the LPC detector, we used the collected dataset as 80% train and 20% test sets for all classes.

### 4.2. Minimum Detectable LP Size

**Figure 11:**
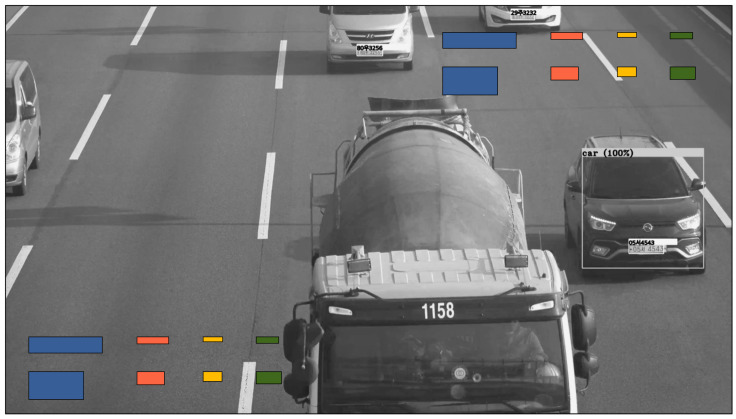
License plate sizes in one-lane, two-lane, three-lane, and four-lane images, represented to scale on 3840 × 2160 resolution.

The KVT-LPR aims to recognize multiple vehicle types and LPs in multilane highways. This means that the size of LPs will be small even in high-resolution images (i.e., 4K resolution) when multiple lanes are observed. To see how our system performs on multilane images, we recorded the detected LPs’ sizes by running test images of one-lane, two-lane, three-lane, and four-lane highways, respectively. The recorded LP sizes were sorted in ascending order of width. For brevity of results, we calculated the average of the first 100 ordered LP sizes. Table 5 shows the smallest 20 LP sizes for each lane with the calculated average. The average LP sizes in the different lanes are visualized in Figure 11.

### 4.3. Detection Speed

**Figure 12:**
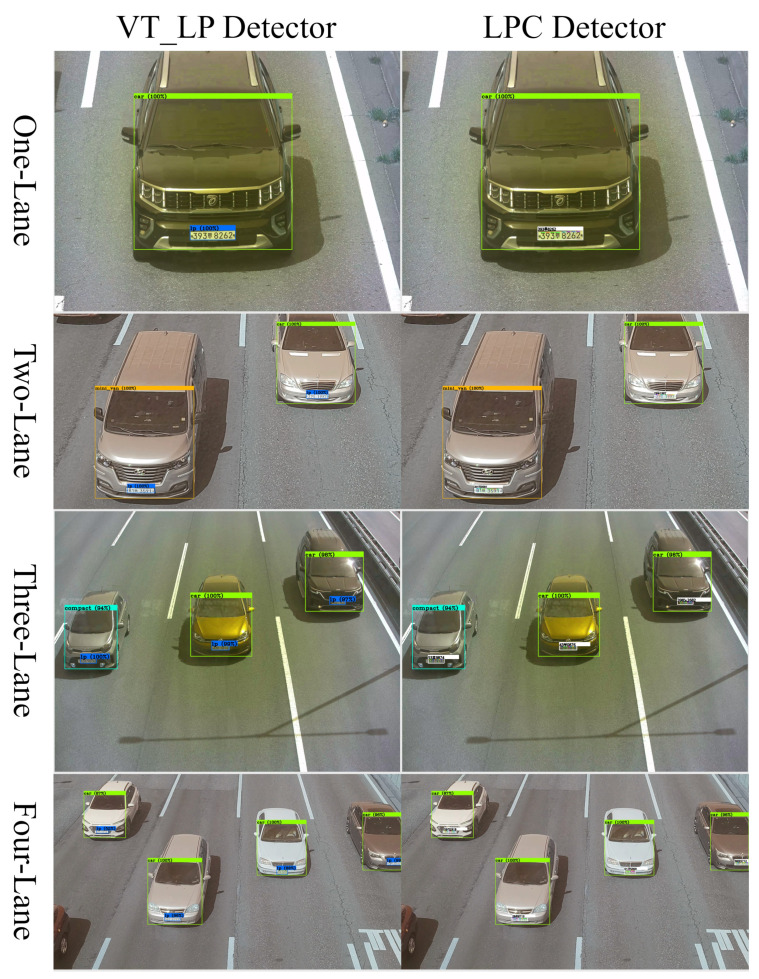
Examples of multilane test images for measuring detection speed.

To measure the detection speed of the KVT-LPR, we used images that contain one car and one LP per lane. This means that one-lane test images (33 images) contained one car and one LP, and two-lane test images (20 images) contained two cars and two LPs. Likewise, three-lane test images (22 images) contained three cars and three LPs, and four-lane test images (19 images) contained four cars and four LPs. Figure 12 shows the examples of test images.

The detection speed is defined as the time it takes to detect vehicle type and LP (phase 1, VT LP detector) and the time it takes to recognize LP characters from a cropped LP image (phase 2, LPC detector). Two platforms running Ubuntu 18.04 were evaluated: a PC with an RTX3090 graphics card (representing a high-end specification, GeForce RTX3090, NVIDIA CUDA Cores 10496, memory 24 GB, AMD Ryzen 7 3700X 8-core processor, 16 GB main memory) and a Jetson AGX Xavier (representing a low-end or embedded specification, 512-core NVIDIA Volta^™^ GPU with 64 tensor cores, 8-core ARM^®^ v8.2 64-bit CPU, 8 MB L2 + 4 MB L3, 32 GB 256-bit LPDDR4x | 137GB/s, 32GB eMMC 5.1). Table 6 and Table 7 show the measured detection speed on two platforms. The detection speed for the VT_LP detector or phase 1 is comparable across different multilanes. However, the detection speed for the LPC detector or phase 2 is significantly reduced. This can be explained by the fact that the VT_LP detector detects only seven classes, whereas the LPC detector detects a magnitude more classes.

### 4.4. Vehicle Type and LP Detection Performance

In the KVT-LPR, the VT_LP detector detects seven classes (LP and six vehicle types). To evaluate the performance of the VT_LP detector, we used typical metrics used for object detection, including precision (Equation (1)), recall (Equation (2)), F1-score (Equation (3)), average IOU (Equation (4)), average precision (Equation (5)), and mAP (Equation (6)).

(1) $$\mathrm{Precision}\;\left(P\right)=\frac{TP}{TP+FP}$$

(2) $$\mathrm{Recall}\;\left(R\right)=\frac{TP}{TP+FN}$$

(3) $$F1-\mathrm{score}=\frac{2\times P\times R}{P+R}$$

(4) $$\mathrm{Average}\;\mathrm{IOU}=\frac{TP}{TP+FP+TN}$$

(5) $$\mathrm{Average}\;\mathrm{Precision}\;\left(\mathrm{AP}\right)=\sum_{n}\left(R_{n}-R_{n-1}\right)P_{n}$$

(6) $$\mathrm{mAP}=\frac{1}{N}\sum_{i=1}^{N}AP_{i}$$

We ran the VT_LP detector with 222 one-lane, 140 two-lane, 183 three-lane, and 133 four-lane test set images, respectively. The vehicle type and LP detection results are shown in Table 8 and Table 9. Our detector stably performed for one-lane, two-lane, and three-lane test images, demonstrating mAPs of 98.0%, 94.0%, and 97.1%, respectively. The most complicated scenario, four-lane, yielded an mAP of 84.6%. The most common cause of failure on three- and four-lane highways is incorrect detection of partially contained vehicles in the upper zone. This can be avoided by pushing the recognition area to the center of the image.

**Figure 13:**
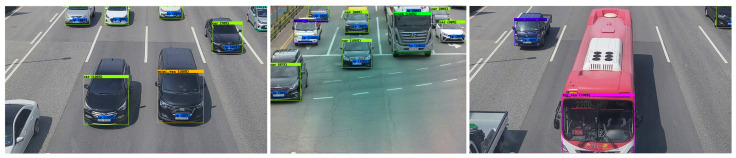
Examples of successfully detected vehicle types (car, mini van, mini truck, truck, bus) with the VT_LP detector in phase 1.

Figure 13 shows examples of successfully detected vehicle types with the VT_LP detector.

### 4.5. License Plate Recognition Performance

**Figure 14:**
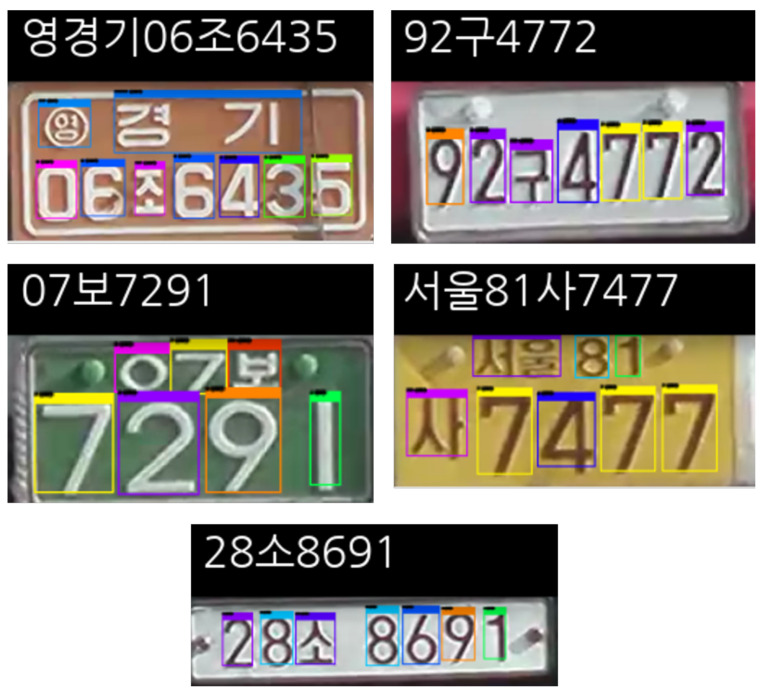
Output examples of the LPC detector in phase 2 using our dataset which reads as `Yeong Gyeonggi 06 Jo 6435’, `92 Gu 4772’, `07 Bo 7291’, `Seoul 81 Sa 7477’, and `28 So 8691’, respectively.

**Figure 15:**
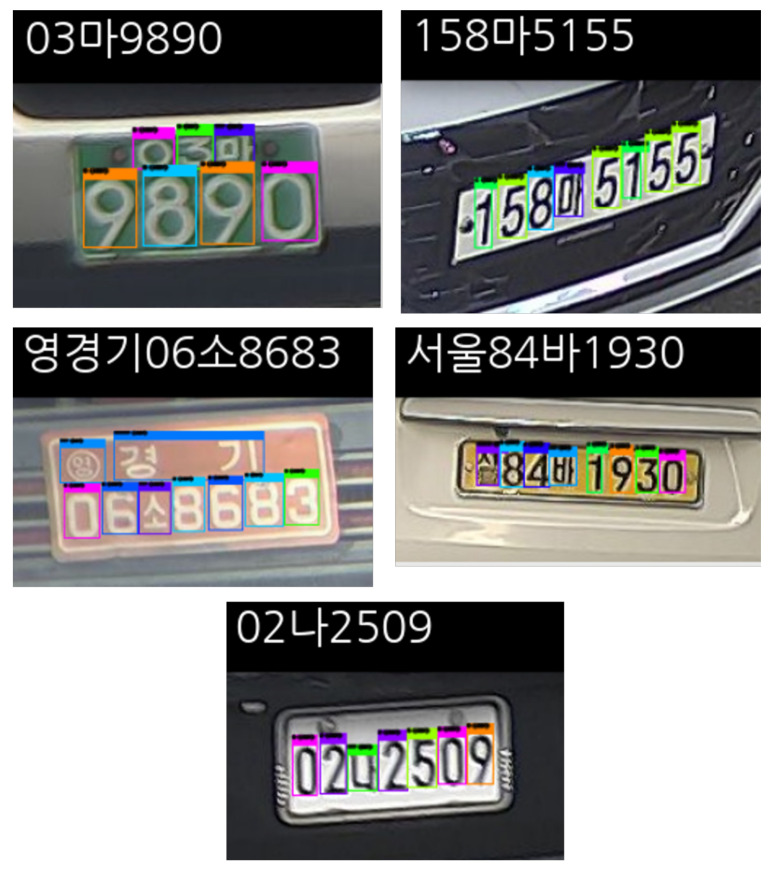
Output examples of the LPC detector in phase 2 using the AI-Hub dataset which reads as `03 Ma 9890’, `158 Ma 5155’, `Yeong Gyeonggi 06 So 8683’, `Seoul 84 Ba 1930’, and `02 Na 2509’.

Phase 2 of the proposed KVT-LPR system was evaluated according to the same metrics. The LPC detector detects 68 classes (i.e., numbers 0 to 9, 17 local area prefixes, and 41 Korean characters). First, we used our custom dataset to evaluate the performance of the LPC detector. As mentioned earlier, our dataset does not include several local area prefixes (i.e., 광주 (Gwangju), 대전 (Daejeon), 세종 (Sejong), 울산 (Ulsan), 전남 (Jeonnam), 전북 (Jeonbuk), 제주 (Jeju) ). Additionally, we used a publicly available LP dataset from AI-Hub (https://aihub.or.kr/aidata/27727, accessed on 7 December 2021). This open dataset includes 100,000 cropped car number plates in JPG format. We excluded local area prefixes not collected in our dataset. We tried to gather another open dataset, such as KarPlate dataset [23], but it was no longer available due to legal issues. There are other approaches, such as synthetically generating LPs [26] and synthetic LP dataset (https://www.idai.or.kr/user/data_market/detail.do?id=63af9c70-ce79-11eb-ba8d-eb1fdd80455f, accessed on 7 December 2021), but we only evaluated our detector with the real data. Figure 14 shows LPR results on our custom dataset. Figure 15 shows LPR results on the AI-Hub dataset.

Table 10 shows the performance of the LPC detector according to the evaluation metrics, and Table 11 shows the detailed per-class results. With relatively few false positives and false negatives, the LPC detector had an adjusted mAP (i.e., eliminating classes with no or sparse data) of 99.30% for our custom dataset and 99.41% for the publicly open AI-Hub dataset.

### 4.6. Discussion

Typical LPR systems use the camera view to monitor and check the LP of a single vehicle. The throughput (i.e., the number of LPs detected) of an LPR system can be enhanced and the deployment cost can be decreased if it can check multiple vehicles in several lanes. The proposed KVT-LPR showed that using multilane high-resolution images for LPR and vehicle type detection is possible. Table 5 shows how our system successfully detected small LP sizes of about 100 pixels. The KVT-LPR can be deployed on an embedded platform such as Jetson AGX. Table 7 shows that a standalone KVT-LPR configuration is feasible, but a networked-system (i.e., sending images to servers for recognition) approach can compensate for its shortcomings.

Our approach has some limitations. First, not all possible Korean LP styles and characters were collected in our dataset. Due to the geographical distance between other regions (i.e., cities and provinces) and our data collection location, several LPs with local area prefixes were left out. More data on those missing locations can be collected to improve our dataset. Second, the vehicle type detection can be improved by disregarding partially visible vehicles in images. In three-lane and four-lane images, those partially visible vehicles often resulted in failure cases. Third, our method assumes that the front view of the vehicle is captured. When the vehicle’s rearview is used for recognition, vehicle type detection using the VT_LP detector is not possible due to this constraint. Regardless, LPR via the LPC detector works in both frontal and rear views. Lastly, as with many previous LPR studies, our dataset is not disclosed for legal reasons (i.e., obtaining the vehicle owner’s consent for distribution and reuse).

## 5. Conclusions

This paper proposed KVT-LPR, a two-phase LPR system based on YOLOv4 for Korean vehicles and LPs. Using 4K high-resolution input images, six vehicle types and LPs are detected by the VT_LP detector, followed by the LPC detector for LPR. The KVT-LPR is applicable to settings (i.e., highly populated and multilane highways in Korea) where the size of LPs is small. Across two datasets (our custom dataset and an open public dataset) and two target systems (RTX3090 and Jetson AGX), two custom detectors in the KVT-LPR demonstrated LPR performance suitable for both high-end and embedded platforms.

Our approach has limitations and drawbacks discussed in previous sections that deserve further research. For example, our dataset can be extended to include national coverage and special purpose vehicles. Moreover, to optimize LPR performance in designated settings (i.e., standalone, over-the-network, on edge devices), various network parameters, including image input size for YOLOv4 or other object detectors, can be compared, and trade-offs can be analyzed. Nonetheless, we have demonstrated the merits of our proposed KVT-LPR to effectively address Korean LPR with vehicle type detection that can be used in various complex smart city applications.

## Figures and Tables

**Table 1:** Comparison of recent Korean LPR systems.

System	Approach	Dataset (Resolution)	Korean LP	Multilanes	Small LP	Vehicle Type	Embedded Platform
Han et al. (2015) [20]	Cascade Structure	Custom (1624 × 1224)	O	X	X	X	X
Park et al. (2019) [21]	KNN	Custom (1920 × 1080)	O	X	X	X	X
Kim et al. (2019) [22]	YOLOv3	UA-DETRAC (960 × 540)	X	O	X	X	X
Henry et al. (2020) [23]	YOLOv3	KarPlate (1920 × 1080)	O	X	X	X	X
Sung et al. (2020) [24]	YOLOv3	KETI-ALPR (3840 × 2160)	O	X	O	X	O
Kim et al. (2020) [25]	Faster-RCNN, YOLOv4, SSD	Custom (N/A)	X	X	X	O	X
Han et al. (2020) [26]	LP-GAN, YOLOv2	Real + Synthetic (N/A)	X	X	X	X	X
Wang et al. (2021) [27]	YOLOv5, WPOD-NET	KarPlate (1920 × 1080)	O	X	O	X	X
Lim and Park (2021) [28]	DNN	CCTV images (N/A)	O	X	X	X	X
Proposed (2021)	Two phase YOLOv4	Custom (3840 × 2160)	O	O	O	O	O

**Table 2:** Our dataset of six vehicle types and LPs with corresponding distribution ratio.

Classes	Multilanes				
	Two	Three	Four	Total	Ratio (%)
License Plate	4063	2411	374	6878	57.1
Car	1757	1159	230	3146	26.1
Mini Truck	563	275	41	879	7.3
Compact	292	170	38	500	4.2
Mini Van	282	97	15	394	3.3
Truck	110	59	12	181	1.5
Bus	48	13	5	66	0.6
Total	7115	4184	715	12,044	100.0

**Table 3:** Collected data for Korean LP numbers.

Class	Character	Training	Validation	Test	Total
0	0	4560	871	686	6117
1	1	4978	966	744	6688
2	2	3850	680	583	5113
3	3	4171	789	608	5568
4	4	3644	657	510	4811
5	5	3775	718	558	5051
6	6	3431	598	510	4539
7	7	3544	710	487	4741
8	8	3449	635	473	4557
9	9	3081	577	434	4092
Total		38,483	7201	5593	51,277

**Table 4:** Collected data for Korean LP characters; classes 55, 57, 60, 61, 63, 64, and 67 were not sufficiently collected (highlighted in gray).

Class	Character	Training	Validation	Test	Total
10	가 (Ga)	137	31	25	193
11	거 (Geo)	150	36	26	212
12	고 (Go)	145	35	27	207
13	구 (Gu)	140	32	24	196
14	나 (Na)	151	35	26	212
15	너 (Neo)	151	35	27	213
16	노 (No)	141	31	26	198
17	누 (Nu)	144	32	26	202
18	다 (Da)	142	32	25	199
19	더 (Deo)	145	35	24	204
20	도 (Do)	142	32	26	200
21	두 (Du)	180	28	21	229
22	라 (Ra)	140	31	25	196
23	러 (Reo)	173	26	20	219
24	로 (Ro)	136	28	21	185
25	루 (Ru)	217	35	27	279
26	마 (Ma)	185	26	20	231
27	머 (Meo)	154	35	27	216
28	모 (Mo)	182	26	20	228
29	무 (Mu)	153	35	27	215
30	바 (Ba)	656	168	120	944
31	배 (Bae)	33	10	6	49
32	버 (Beo)	141	32	26	199
33	보 (Bo)	164	19	16	199
34	부 (Bu)	163	15	13	191
35	사 (Sa)	106	27	21	154
36	서 (Seo)	126	11	9	146
37	소 (So)	124	12	9	145
38	수 (Su)	115	13	11	139
39	아 (A)	259	52	46	357
40	어 (Eo)	115	18	15	148
41	영 (Yeong)	54	12	9	75
42	오 (O)	124	17	15	156
43	우 (U)	132	8	8	148
44	자 (Ja)	264	53	37	354
45	저 (Jeo)	116	14	12	142
46	조 (Jo)	115	16	11	142
47	주 (Ju)	137	13	10	160
48	하 (Ha)	125	15	13	153
49	허 (Heo)	117	17	17	151
50	호 (Ho)	134	32	19	185
51	강원 (Gangwon)	136	35	22	193
52	경기 (Gyeonggi)	187	44	38	269
53	경남 (Gyeongnam)	106	25	16	147
54	경북 (Gyeongbuk)	51	11	9	71
55	광주 (Gwangju)	5	0	0	5
56	대구 (Daegu)	98	22	16	136
57	대전 (Daejeon)	0	0	0	0
58	부산 (Busan)	20	5	3	28
59	서울 (Seoul)	283	67	52	402
60	세종 (Sejong)	0	0	0	0
61	울산 (Ulsan)	3	0	0	3
62	인천 (Incheon)	165	39	29	233
63	전남 (Jeonnam)	5	0	1	6
64	전북 (Jeonbuk)	3	0	0	3
65	충남 (Chungnam)	331	76	60	467
66	충북 (Chungbuk)	11	1	1	13
67	제주 (Jeju)	0	0	0	0
Total		7832	1535	1180	10,547

**Table 5:** Successfully detected smallest LPs in ascending order of their widths.

	One-Lane				Two-Lane				Three-Lane				Four-Lane			
	**Single-Line LP**		**Double-Line LP**		**Single-Line LP**		**Double-Line LP**		**Single-Line LP**		**Double-Line LP**		**Single-Line LP**		**Double-Line LP**	
	**Width**	**Height**	**Width**	**Height**	**Width**	**Height**	**Width**	**Height**	**Width**	**Height**	**Width**	**Height**	**Width**	**Height**	**Width**	**Height**
1	286	94	250	137	149	49	119	72	96	32	102	36	113	36	106	59
2	309	34	258	149	159	48	125	70	96	25	102	44	113	36	106	43
3	364	90	258	137	164	40	133	75	97	32	102	59	115	35	106	51
4	372	54	261	140	164	34	133	65	99	22	102	54	115	33	108	52
5	372	87	262	138	165	36	133	77	99	26	102	50	118	35	108	58
6	374	96	262	143	165	38	134	75	100	30	102	44	118	32	111	44
7	378	90	266	126	166	38	136	65	100	25	102	50	119	35	112	65
8	382	35	267	133	166	44	137	75	100	26	102	46	119	37	116	54
9	384	81	270	142	166	27	137	68	101	27	103	55	119	35	116	67
10	384	89	270	147	166	37	137	70	101	26	103	53	119	35	117	62
11	384	96	272	119	167	32	139	73	101	27	103	41	120	37	117	54
12	387	52	273	118	167	38	140	68	101	26	103	55	120	27	118	44
13	388	89	274	153	168	33	140	70	101	16	103	52	121	35	119	75
14	391	95	276	156	169	34	140	67	102	32	103	56	121	34	119	58
15	391	89	278	155	169	41	140	69	102	33	103	37	121	35	119	60
16	392	84	278	156	170	39	140	63	102	29	103	54	121	37	122	75
17	393	98	280	136	170	35	141	75	104	27	103	57	121	34	122	65
18	393	91	280	143	170	35	141	68	104	21	103	52	121	36	123	64
19	395	95	280	151	170	40	142	90	104	28	103	51	121	29	124	71
20	395	65	280	152	171	38	143	71	104	27	103	48	122	32	124	58
Average	408	89	304	155	176	39	153	73	108	27	105	54	126	35	140	69

**Table 6:** Detection speed on a PC with RTX3090.

	One-Lane		Two-Lane		Three-Lane		Four-Lane	
	Elapsed Time (ms)	FPS	Elapsed Time (ms)	FPS	Elapsed Time (ms)	FPS	Elapsed Time (ms)	FPS
VT_LP detector	7.16	139.71	7.28	137.34	7.99	125.08	8.33	120.10
LPC detector	17.70	56.51	29.81	33.55	44.22	22.62	54.42	18.38

**Table 7:** Detection speed on Jetson AGX.

	One-Lane		Two-Lane		Three-Lane		Four-Lane	
	Elapsed Time (ms)	FPS	Elapsed Time (ms)	FPS	Elapsed Time (ms)	FPS	Elapsed Time (ms)	FPS
VT_LP detector	60.68	16.48	61.74	16.20	63.35	15.79	64.25	15.56
LPC detector	133.44	7.49	220.26	4.54	309.44	3.23	375.08	2.67

**Table 8:** Vehicle type and LP detection, mAP at IOU = 0.5.

Class	Average Precision			
	One-Lane	Two-Lane	Three-Lane	Four-Lane
License Plate	99.4	99.9	98.1	95.2
Car	99.9	93.9	94.5	95.0
Mini Truck	99.4	99.6	97.2	98.4
Compact	87.5	75.0	98.1	75.0
Mini Van	99.4	90.0	94.6	69.4
Truck	100.0	100.0	100.0	75.0
Bus	100.0	100.0	100.0	100.0
mAP	98.0	94.0	97.1	84.6

**Table 9:** Performance of the VT_LP detector.

	One-Lane	Two-Lane	Three-Lane	Four-Lane
Precision	0.98	0.92	0.92	0.87
Recall	0.99	0.97	0.98	0.94
F1-score	0.99	0.95	0.95	0.90
Average IOU (%)	90.3	87.5	90.8	77.8

**Table 10:** Performance of the LPC detector.

	Our Dataset	AI-Hub Dataset
Precision	0.99	1.00
Recall	1.00	1.00
F1-Score	0.99	1.00
Average IOU (%)	92.84	99.41

**Table 11:** Performance of the LPC detector per class; classes highlighted in gray are excluded for adjusted results.

Class	Our Dataset			AI-Hub Dataset		
	AP	TP	FP	AP	TP	FP
0	99.65	868	2	99.93	4470	3
1	99.82	965	14	99.94	5538	11
2	99.83	679	4	99.83	5051	26
3	99.87	788	4	99.95	5599	9
4	99.40	654	3	99.76	4451	5
5	98.68	718	8	98.80	4724	4
6	99.83	597	0	99.87	4742	5
7	99.86	709	9	99.80	4421	22
8	100.00	635	0	99.94	4824	6
9	99.83	575	1	99.86	4186	0
가 (Ga)	100.00	31	2	99.90	198	0
거 (Geo)	97.07	34	0	98.49	197	5
고 (Go)	100.00	35	1	99.00	198	0
구 (Gu)	100.00	32	0	97.50	195	0
나 (Na)	97.14	34	0	100.00	199	0
너 (Neo)	99.68	35	1	99.99	200	3
노 (No)	96.77	30	1	98.96	197	2
누 (Nu)	100.00	32	0	98.51	198	0
다 (Da)	100.00	32	0	99.49	199	2
더 (Deo)	97.06	33	0	96.50	193	0
도 (Do)	100.00	32	2	98.50	197	3
두 (Du)	100.00	28	0	99.85	200	10
라 (Ra)	100.00	31	0	100.00	198	0
러 (Reo)	100.00	26	0	99.47	196	6
로 (Ro)	89.70	25	11	96.26	193	2
루 (Ru)	92.14	32	9	98.92	198	13
마 (Ma)	100.00	26	0	99.00	198	1
머 (Meo)	99.86	35	0	96.05	195	4
모 (Mo)	100.00	25	0	98.79	197	3
무 (Mu)	100.00	35	0	98.17	197	1
바 (Ba)	100.00	168	1	98.80	249	2
배 (Bae)	100.00	10	0	0	0	0
버 (Beo)	100.00	32	0	99.99	200	4
보 (Bo)	100.00	19	0	99.99	200	4
부 (Bu)	100.00	15	0	99.99	201	5
사 (Sa)	99.87	26	0	93.73	198	2
서 (Seo)	100.00	11	0	99.98	200	22
소 (So)	100.00	12	0	99.99	199	4
수 (Su)	100.00	13	0	99.99	200	4
아 (A)	100.00	52	0	97.13	203	0
어 (Eo)	100.00	18	0	96.96	193	3
영 (Yeong)	98.81	11	2	0	0	0
오 (O)	100.00	17	0	97.66	198	17
우 (U)	100.00	8	0	98.99	197	1
자 (Ja)	100.00	53	0	90.04	208	0
저 (Jeo)	100.00	14	0	97.84	198	25
조 (Jo)	100.00	16	0	100	201	0
주 (Ju)	100.00	13	0	98.48	195	0
하 (Ha)	100.00	15	0	99.48	200	2
허 (Heo)	100.00	17	0	99.31	198	7
호 (Ho)	100.00	32	0	89.59	178	1
강원 (Gangwon)	97.14	34	0	0	0	0
경기 (Gyeonggi)	100.00	44	3	96.36	159	0
경남 (Gyeongnam)	99.70	24	1	0	0	0
경북 (Gyeongbuk)	100.00	11	0	0	0	0
광주 (Gwangju)	0	0	0	0	0	0
대구 (Daegu)	100.00	22	0	0	0	0
대전 (Daejeon)	0	0	0	0	0	0
부산 (Busan)	100.00	5	0	0	0	0
서울 (Seoul)	99.98	66	1	97.83	724	2
세종 (Sejong)	0	0	0	0	0	0
울산 (Ulsan)	0	0	0	0	0	0
인천 (Incheon)	100.00	39	0	0	0	0
전남 (Jeonnam)	0	0	0	0	0	0
전북 (Jeonbuk)	0	0	0	0	0	0
충남 (Chungnam)	96.00	74	3	0	0	0
충북 (Chungbuk)	100.00	1	0	0	0	0
제주 (Jeju)	0	0	0	0	0	0
Total	89.08	8703	83	73.87	56,648	251
Adjusted	99.30	8703	83	99.41	56,648	251
